# Branched-Chain Amino Acids Are Neuroprotective Against Traumatic Brain Injury and Enhance Rate of Recovery: Prophylactic Role for Contact Sports and Emergent Use

**DOI:** 10.1089/neur.2022.0031

**Published:** 2022-08-16

**Authors:** Rob D. Dickerman, Julie Williamson, Ezek Mathew, Christopher M. Butt, Clark W. Bird, Lauren E. Hood, Vivian Grimshaw

**Affiliations:** ^1^ Department of Neurosurgery, University of North Texas Health Science Center (UNTHSC), Frisco, Texas, USA.; ^2^ Department of Neuroscience, Inotiv-Boulder, Inc., Boulder, Colorado, USA.

**Keywords:** branched-chain amino acids (BCAAs), concussion, glutamate, neuroprotective, traumatic brain injury

## Abstract

Branched-chain amino acids (BCAAs) are known to be neurorestorative after traumatic brain injury (TBI). Despite clinically significant improvements in severe TBI patients given BCAAs after TBI, the approach is largely an unrecognized option. Further, TBI continues to be the most common cause of morbidity and mortality in adolescents and adults. To date, no study has evaluated whether BCAAs can be preventive or neuroprotective if taken before a TBI. We hypothesized that if BCAAs were elevated in the circulation before TBI, the brain would readily access the BCAAs and the severity of injury would be reduced. Before TBI induction with a standard weight-drop method, 50 adult mice were randomized into groups that were shams, untreated, and pre-treated, post-treated, or pre- + post-treated with BCAAs. Pre-treated mice received BCAAs through supplemented water and were dosed by oral gavage 45 min before TBI induction. All mice underwent beam walking to assess motor recovery, and the Morris water maze assessed cognitive function post-injury. On post-injury day 14, brains were harvested to assess levels of astrocytes and microglia with glial fibrillary acidic protein (GFAP) and ionized calcium-binding adapter molecule 1 (IBA-1) immunohistochemistry, respectively. Pre-treated and pre- +post-treated mice exhibited significantly better motor recovery and cognitive function than the other groups. The pre- + post-treated group had the best overall memory performance, whereas the pre-treated and post-treated groups only had limited improvements in memory compared to untreated animals. Pre- + post-treated brains had levels of GFAP that were similar to the sham group, whereas the pre-only and post-only groups showed increases. Although trends existed, no meaningful changes in IBA-1 were detected. This is the first study, animal or human, to demonstrate that BCAA are neuroprotective and substantiates their neurorestorative benefits after TBI, most likely through the important roles of BCAAs to glutamate homeostasis.

## Introduction

Traumatic brain injury (TBI) remains the most common cause of morbidity and mortality in adolescents and adults, with ∼13 million persons affected annually among the United States and Europe.^1^ Sports-related TBI is estimated to be >4 million annually, with up to 85% of TBIs classified as mild, and the majority of mild concussions are never reported.^2^ Kara and colleagues demonstrated that less than half of patients suffering from sports-related concussion recover within 2 weeks and most require up to 28 days.^3^ A recent study on mild TBI demonstrated that patients can have elevated inflammatory cytokines up to 12 months after injury.^2^ Thus, early intervention for reversal of the complex, non-linear pathobiochemical cascade is essential to halt or reverse damage.

The pathophysiology of TBI occurs in phases with a progressive cascade of events.^4^ The first step in this cascade occurs in the acute phase (<1 h), involves the immediate and massive release of glutamate from the pre-synaptic terminals, and disrupts the ionic equilibrium of post-synaptic membranes. Glutamate, being the major excitatory neurotransmitter, leads to rapid depolarization of post-synaptic membranes,^5^ and the amount of glutamate released correlates with the severity of injury and with mortality.^6^ Extracellular potassium release depends on the initial release of glutamate. As extracellular potassium increases, this leads to increases in intracellular calcium and subsequent increases in mitochondrial uptake of calcium. Excess mitochondrial calcium leads to oxidative stress, which impairs mitochondrial function and can lead to cellular death. Interestingly, accumulation of intracellular calcium has been correlated with cognitive dysfunction, and, as calcium levels drop, cognitive function improves. It has been shown that older animals lose the ability to reverse the calcium accumulation, which leads to cell death.^4^ Given that glutamate is the key in the initial cascade of events, reversing the excess glutamate released after trauma, or rapidly lowering glutamate levels, could stop the cascade.

Branched-chain amino acids (BCAAs) in normal physiological conditions function as substrates for protein synthesis, but also help regulate protein and energy metabolism. An important neurological role for BCAA is as a nitrogen donor for 30–50% of glutamate synthesis. Animal and human studies have both demonstrated beneficial effects of (BCAA) after TBI.^7–9^ Cole and colleagues demonstrated that providing BCAA in drinking water after TBI restored hippocampal levels of BCAA and improved cognitive function as compared to untreated TBI mice. Moreover, sham mice receiving BCAA did not show elevations in hippocampal BCAA levels, indicating that BCAA utilization in the brain is dependent on its current need for BCAA.^7^

Human studies on TBI are more difficult to perform because of the heterogeneity of the brain injuries, the complexity of identifying matched controls, and differences in the standard of care at multiple study sites. Jeter and colleagues measured circulating BCAA levels in normal volunteers and in mild and severe TBI patients. They found that BCAA levels were significantly lower in TBI patients, and lower BCAA levels correlated with the severity of TBI, suggesting that BCAAs were being utilized by the brain.^8^ Aquilani and colleagues performed a study on severe TBI patients and provided a 19-g daily dose of BCAA to 20 severe TBI patients and compared them to 20 age-matched and Glasgow Coma Scale–matched controls. Impressively, 68% of BCAA-treated patients emerged from vegetative states, whereas none of the untreated changed (*p* < 0.0001).^9^ In 2021, updated neurosurgical guidelines for the management of severe TBI were published. We commented on the need for revising the nutritional component of these guidelines and recommended BCAA for TBI patients.^10^ Our hypothesis is that if circulating BCAAs are elevated at the time of TBI, the BCAAs will prevent or reduce the severity of TBI.

## Methods

### Animals

Adult, female C57BL/6J mice were obtained from Jackson Labs, Inc. (Bar Harbor, ME) and housed in groups of 5 on a 12 h/12 h light schedule with *ad libitum* access to chow and water throughout the study. The ordering and receipt of the animals were coordinated with the study design that called for 3 days of pre-treatment before injury and a standard injury weight of 20.0 ± 0.5 g. All animals were between 84 and 91 days of age at the time of injury. There was a total of 50 mice with *N* = 10 per group. All animal procedures were approved by the Institutional Animal Care and Use Committee at Inotiv-Boulder, Inc. (Boulder, CO). At the time these experiments were conducted, only female mice had been validated.

### Study design

A pilot study determined peak plasma leucine levels in these mice at baseline and at 30, 45, 90, and 120 min after a single oral gavage dose of BCAA (isoleucine, 168 mg/kg; leucine, 335 mg/kg; valine, 168 mg/kg; 20 mL/kg dose volume). Dosing was based on an equivalent human dose of 21 g of BCAA in a 2:1:1 ratio.^11^ Leucine levels were measured by liquid chromatography/mass spectroscopy and levels peaked at 45 min after dosing (data not shown).

Treatments (*ad libitum* water or *ad libitum* water supplemented with BCAA (isoleucine, 1.25 g/kg/d; leucine, 2.5 g/kg/d; valine, 1.25 g/kg/d) were provided for 3 days before a sham procedure or TBI (see below). In accordance with the pre-determined plasma absorption profile of leucine after oral gavage 45 min directly before sham or TBI induction, all animals received either water or water supplemented with BCAA (isoleucine, 168 mg/kg; leucine, 335 mg/kg; valine, 168 mg/kg; 20-mL/kg dose volume) by oral gavage. Directly after sham or TBI induction, animals were either maintained on water alone (sham or TBI), switched to water alone (pre-injury BCAA; Pre), or switched to BCAA-supplemented water (post-injury BCAA; Post and Pre + Post) at the doses described above for *ad libitum* administration. Animals were randomized to the groupings by weight, and the researchers were blinded from the treatments throughout all experimental procedures.

### Weight-drop injury

Fifty mice were anesthetized with 2.5% isoflurane delivered for 2 min. They were then placed individually in the prone position on a foam bed with heads directly under a Plexiglas tube. A brass weight (80 g) was dropped once through the tube from 0.8 m, striking the head directly and resulting in a closed-head injury. Animals were moved directly after being struck by the weight so that no secondary impacts occurred. They were then provided buprenorphine (0.1 mg/kg subcutaneously) directly after injury. Sham-injured mice underwent the same procedure, but the weight was not released. All mice were placed in a heated recovery cage and monitored until ambulatory (∼5–15 min) before being returned to their cage. Sham-injured mice recovered the righting reflex at 24.3 ± 4.8 sec, whereas injured mice had righting reflex recovery times >2 min. Based upon these righting reflex times, all injured groups were comparable (*F*_(3,27)_ = 1.03, *p* = 0.39; pre-treated, 129.3 ± 21.2 sec; post-treated, 316.9 ± 124.0 sec; Pre- + Post-treated, 244.0 ± 91.2 sec; TBI only, 226.5 ± 27.2 sec). In addition, daily post-procedure monitoring indicated that all animals that initially survived TBI induction had normal home cage behavior and no unexpected changes in body weight or water consumption.

### Beam walking

A narrow wooden beam (5 mm wide × 1 m long) was suspended 1 m above the ground with a goal box at the end. The animal was placed on the beam, and the number of foot faults for the right hindlimb was recorded over 50 steps. A baseline level of performance was achieved after 3 days of training (four trials per day) before TBI, and animals were tested at 1 and 3 days post-TBI (one trial each day) to assess acute motor recovery.

### Morris water maze

Spatial learning ability was assessed in all mice 4 days after TBI induction. On days 4–11 after injury or sham, mice were trained with four trials per day and a 15-min intertrial interval. Mice started from one of the four pool quadrants, and the starting point was varied for each animal and each testing day. If an animal did not find the platform within 60 sec during a given trial, it was placed on the platform for 10 sec by the handler. Four hours after the last acquisition trial, the platform was removed, and a 60-sec retention trial was performed. Twenty-four hours after the last acquisition trial, the platform was removed, and a 60-sec, longer-term retention trial was performed. Every trial was recorded and analyzed with EthoVision 15 (Noldus Inc., Wageningen, The Netherlands).

### Necropsy analysis

On day 14 of the study, mice were euthanized and the resulting brains were fixed in 4% paraformaldehyde. Brains were then sliced and processed for immunohistochemistry for glial fibrillar acidic protein (GFAP; astrocyte marker) and ionized calcium-binding adaptor molecule 1 (IBA-1; microglia marker). GFAP was detected using Abcam anti-GFAP (EPR1034Y; 1:200; Abcam, Cambridge, MA) that was visualized by reacting a horseradish peroxidase–conjugated secondary polymer reacted with diaminobenzidine. IBA-1 was detected using Abcam anti-iba-1 (EPR16588; 1:15,000) that was visualized with the Bond Polymer Refine Red Detection Kit (Leica DS9390; Leica Biosystems, Wetzlar, Germany). A hematoxylin counterstain was used to visualize nuclei. Percent area analysis (Visiopharm, Hoersholm, Denmark) was then used to quantitate the extent of GFAP and IBA-1 expression in the hippocampus and cerebral cortex (three slices per animal; *N* = 5 for each group).

### Statistical analysis

Some animals were lost at TBI induction because of skull fracture or brain hemorrhage (TBI only, 2; pre-treated, 2; post-treated, 3; pre- + post-treated, 3; sham, 1 because of accidental TBI induction and subsequent death). Two additional shams were removed from the water-maze data because they did not reach the learning criteria for normal animals (>50% reduction in escape latency time). Thus, the sample sizes in the behavioral measures were reduced accordingly. Groups were compared using one-way analysis of variance (ANOVA) or one-way repeated-measures ANOVA (RMANOVA), followed by Tukey's *post hoc* tests, to determine group differences. Significance for all tests was set at *p* < 0.05. All graphing and statistical tests were performed with GraphPad Prism (v5.0; GraphPad Software, San Diego, CA).

## Results

### Acute motor recovery

**FIG. 1.**
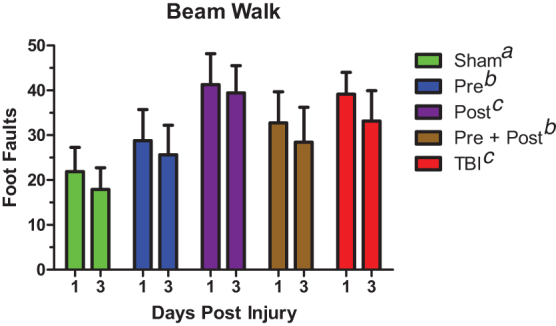
Pre-treatment with BCAAs improved acute motor recovery after TBI. Animals provided BCAAs before injury (Pre and Pre + Post) had better beam walking performance than untreated animals and animals given BCAA after injury alone (Post). Error bars represent the SEM. *N* = 7–9 per group at each time point. Groups that do not share common letter designations differed significantly in Tukey's *post hoc* test. BCAA, branched-chain amino acid; SEM, standard error of the mean; TBI, traumatic brain injury.

Figure 1 shows that BCAA pre-treatment was necessary to improve performance in the beam walking task after TBI on post-injury days 1 and 3. One-way RMANOVA detected a main effect of treatment (*F*_(1,4)_ = 107.9, *p* < 0.001), whereas Tukey's *post hoc* test indicated that pre- and pre- + post-treated animals had significantly fewer foot faults than untreated TBI animals and animals treated only after TBI.

### Spatial learning and memory

**FIG. 2.**
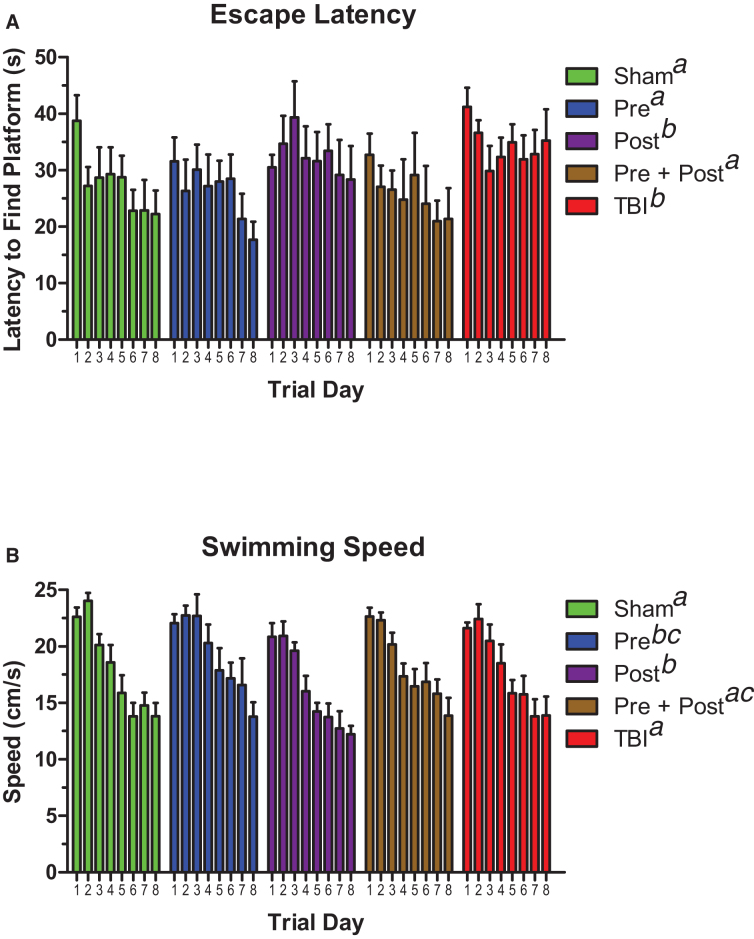
Pre-treatment with BCAAs improved spatial learning after TBI better than post-treatment alone. (**A**) Animals provided BCAAs before the injury (Pre and Pre + Post) had better task learning in the Morris water maze than untreated animals and animals given BCAAs after the injury alone (Post). (**B**) Effects of BCAA treatment on swimming speed. Sham, TBI, and Pre + Post animals were equivalent. Pre-treated animals were faster than shams and TBI animals, but no other groups. Post-treated animals were slower than sham, TBI, and Pre + Post animals, and Pre- + Post-treated animals were only faster than Post-treated animals. Error bars represent the SEM. *N* = 7–8 per group. Data sets that do not share common letter designations differed significantly in Tukey's *post hoc* test. BCAA, branched-chain amino acid; SEM, standard error of the mean; TBI, traumatic brain injury.

Latency to find the submerged platform in the Morris water maze was measured to assess spatial learning acquisition on days 4–11 post-injury. As Figure 2A illustrates, one-way RMANOVA detected a main effect of treatment (*F*_(4,28)_ = 12.17, *p* < 0.001), and, over the course of the task training, the sham, pre-treated, and pre- + post-treated groups found the platform significantly faster than the untreated TBI group and the animals that received post-treatment alone. Swimming speed (Fig. 2B) was also affected by treatment (*F*_(4,28)=_ 12.97, *p* < 0.001), but the pattern of significant group differences was not fully consistent with escape latency or acute motor recovery. However, post-treated animals were slower than sham, pre + post, and TBI animals, even though they had speeds that were equivalent to pre-treated animals.

**FIG. 3.**
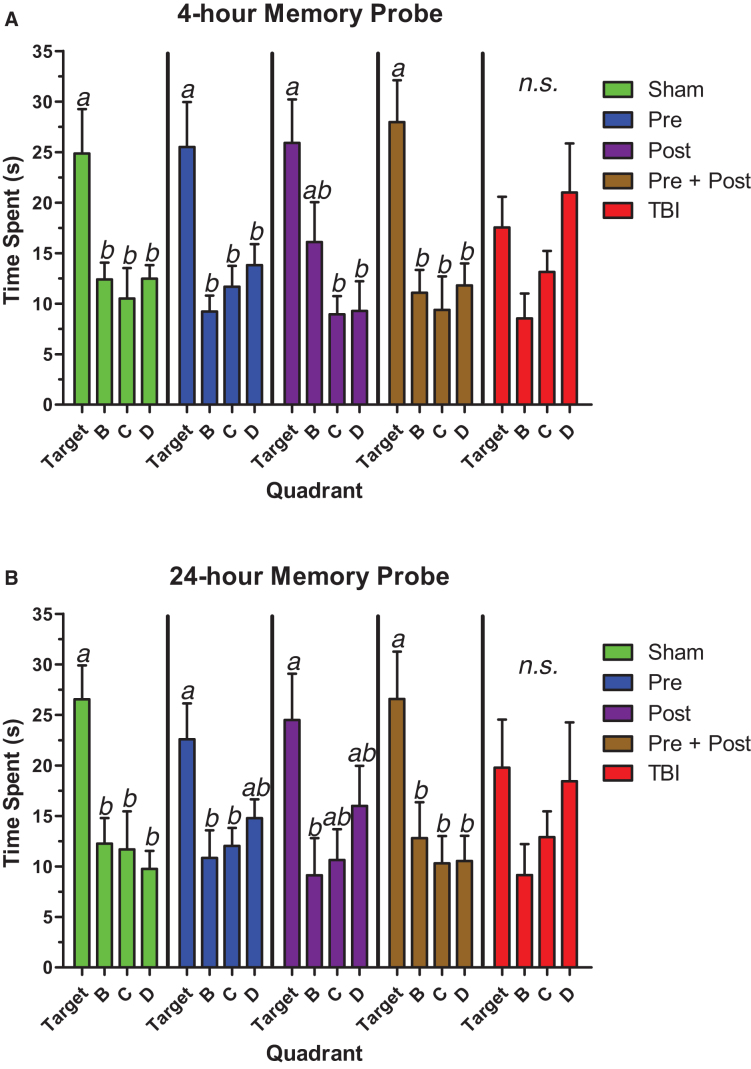
Pre-treatment with BCAAs improved spatial memory after TBI better than post-treatment alone. (**A**) Animals provided BCAAs before the injury (Pre and Pre + Post) remembered the location of the escape platform in a short-term, 4-h memory task more effectively than untreated animals and animals given BCAAs after the injury alone (Post). (**B**) Animals provided BCAAs both before and after brain injury (Pre + Post) remembered the location of the escape platform in a longer-term, 24-h memory task more effectively than untreated animals (TBI), pre-treated animals (Pre), and animals given BCAAs after injury alone (Post). Error bars represent the SEM. *N* = 7–8 per group. Data sets that do not share common letter designations differed significantly in Tukey's *post hoc* test. BCAA, branched-chain amino acid; SEM, standard error of the mean; TBI, traumatic brain injury.

The same group differences persisted in the short-term, 4-h memory probe trials when the escape platform was removed (Fig. 3A). Shams, pre-treated animals, and pre- + post-treated animals were able to differentiate the target quadrant of the maze from all other maze quadrants, as assessed by one-way ANOVA followed by Tukey's *post hoc* test. In contrast, the untreated TBI group could not differentiate any of the quadrants, whereas the post-treated group had some memory but could only differentiate quadrants on the opposite side of the maze (quadrants C and D) from the target quadrant. However, a main effect on quadrant time was still detected in the post-treated group. The statistical parameters were as follows: shams, *F*_(3,24)_ = 5.30, *p* = 0.006; pre-treated, *F*_(3,24)_ = 6.70, *p* = 0.002; post-treated, *F*_(3,24)_ = 5.52, *p* = 0.005; pre- + post-treated, *F*_(3,24)_ = 7.96, *p* < 0.001; untreated TBI, not significant (*F*_(3,28)_ = 2.71, *p* = 0.064).

Figure 3B shows that the results of the longer-term, 24-h memory probe trials were similar to those of the short-term probes, but the pre-treated animals did not perform as well. Only the shams and pre- + post-treated animals differentiated the target quadrant from the other quadrants, whereas the pre-treated animals and post-treated animals could tell the difference between the target and only two of the three non-target quadrants. Again, the untreated TBI group had no preference for any particular quadrant. The statistical parameters were as follows: shams, *F*_(3,24)_ = 6.77, *p* = 0.002; pre-treated, *F*_(3,24)_ = 4.16, *p* = 0.017; post-treated, *F*_(3,24)_ = 3.22, *p* = 0.040; pre- + post-treated, *F*_(3,24)_ = 5.02, *p* = 0.008; untreated TBI, not significant (*F*_(3,28)_ = 1.35, *p* = 0.278).

### Glial fibrillary acidic protein and ionized calcium-binding adapter molecule 1 immunohistochemistry

**FIG. 4.**
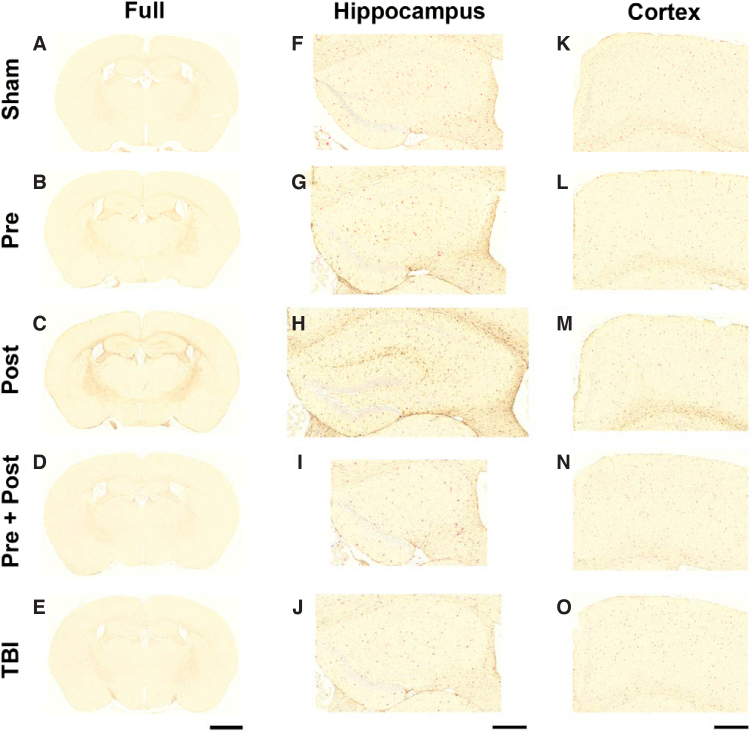
Timing of BCAA treatment affected markers for microglia (IBA-1; red) and astrocytes (GFAP; brown) post-TBI. Representative coronal sections from (**A**) sham mice, (**B**) pre-treated mice, (**C**) post-treated mice, (**D**) Pre- + Post-treated mice, and (**E**) untreated TBI mice. All brain-injured groups exhibited focal regions of increased IBA-1 and GFAP, particularly the hippocampus (increased magnification in panels **F–J**) and cortex (increased magnification in panels **K–O**), whereas uninjured sham mice displayed minimal levels of both markers. Scale bar for panels A through E = 1.6 mm. Scale bars for panels F through J and K through O = 400 μm. BCAA, branched-chain amino acid; GFAP, glial fibrillary acidic protein; IBA-1, ionized calcium-binding adapter molecule 1; TBI, traumatic brain injury.

The effect of BCAA on TBI-induced markers for microglia (IBA-1) and astrocytes (GFAP) has not been evaluated previously. Figure 4 suggests a trend for TBI increasing the expression of GFAP, and BCAA treatment modified those increases depending on when those treatments were delivered. However, the protective effect of BCAA was most evident in animals treated both before and after TBI (Pre + Post). Uninjured sham rats displayed minimal levels of IBA-1 and GFAP, consistent with the fact that microglia and astrocytes reside normally in the brain (Fig. 4A). Conversely, all brain-injured groups exhibited regions of increased IBA-1 and GFAP, particularly in the cortex, hippocampus, corpus callosum, and internal capsule. All of these brain regions are in the path of the TBI-induced force wave that first passes through the sagittal midline of the brain from superior to inferior (Figs. 4B–E). The hippocampus (Figs. 4F–J) and cortex (Figs. 4K–O) were evaluated further at higher magnification. Pre-treatment with BCAA (Pre) appeared to reduce TBI-induced microglia, but upregulated TBI-induced astrocytes. Post-injury treatment with BCAA (Post) also had a trend of reduced TBI-related microglia. However, post-treatment also upregulated TBI-related astrocytes to a greater degree than observed with pre-treatment.

Importantly, the combination of pre- + post-treatment with BCAA normalized the expression of TBI-induced IBA-1 to levels that were equivalent to shams, and the combination did not upregulate levels of GFAP. In both regions, TBI alone appeared to cause some changes in microglia and astrocytes, but these changes were minimal.

**FIG. 5.**
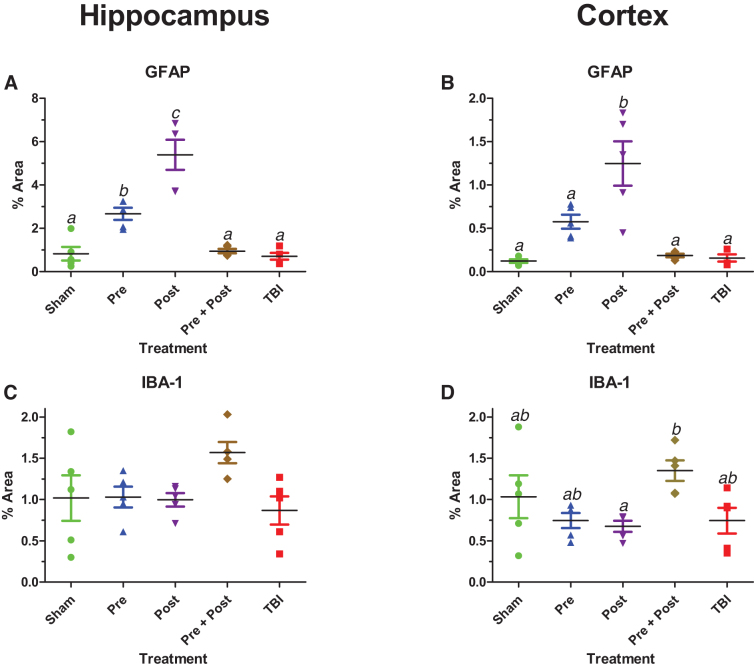
Quantification of GFAP (**A,B**) and IBA-1 (**C,D**) by percent area analysis in the hippocampus and cortex. Data from each animal are plotted with black bars representing the group mean and the correspondingly colored error bars representing the SEM. Data sets that differed significantly in Tukey's *post hoc* tests do not share any letters. GFAP, glial fibrillary acidic protein; IBA-1, ionized calcium-binding adapter molecule 1; SEM, standard error of the mean.

Many of the qualitative trends were confirmed by quantitation of GFAP and IBA-1 in the hippocampus and cortex by percent area analysis (Fig. 5). One-way ANOVA detected a main effect of treatment on GFAP expression in both brain regions (*F*_(4,20)_ = 29.10, *p* < 0.001 in hippocampus, Fig. 5A; *F*_(4,20)_ = 15.39, *p* < 0.001 in cortex, Fig. 5B). Pre-injury treatment with BCAA increased GFAP levels in both brain regions, but this increase was statistically significant only in the hippocampus. Post-injury treatment with BCAA induced significant increases of GFAP in both regions. Combination treatment (Pre + Post) resulted in GFAP levels that were equivalent to those in sham animals. Effects of TBI alone were not significant as determined by Tukey's *post hoc* tests. Quantification of IBA-1 in both brain regions largely did not detect any effect of treatment or TBI (*F*_(4,20)_ = 2.55, *p* = 0.071 in hippocampus; Fig. 5C). Although trends were apparent, the only significant differences in IBA-1 levels were between post-treated animals and combination-treated animals in the cortex (Fig. 5D; *F*_(4,20)_ = 3.32, *p* = 0.031).

## Discussion

This is the first study, animal or human, to demonstrate that prophylactic BCAAs are neuroprotective. Treatment with BCAA clearly provided a protective effect to both motor and cognitive function after TBI. In addition, previous work provides further support to the findings reported here and explains the BCAA mechanisms of action.

The study methods and design were based on previously reported TBI literature.^12–14^ Tucker and colleagues did a comparative TBI study on male and female mice and found that both sexes responded to TBI similarly, suggesting that hormone cycling has limited effects on functional impairments after TBI even though exogenous progesterone is neuroprotective after TBI.^12,13^ Although no animal model can provide a perfect replica of the complex pathobiochemical cascade that occurs in human TBI, the experimental TBI murine model we utilized was recently published as providing a suitable model to replicate human TBI histopathology.^14^

Second, numerous animal studies have shown that BCAAs have a neurorestorative effect after TBI, but the behavioral tests used in this study indicated that pre-treated and pre-/post-treated groups performed better than post-treated animals.^7,8,12,14–17^ In the case of the motor function, beam walk testing was performed only on post-injury days 1 and 3, suggesting that post-treated mice did not have enough exposure time for the beneficial effects of BCAAs. This assertion is supported by the fact that at least 5 days of post-injury treatment with BCAAs was required to elicit protective effects on pain-/fear-conditioned memory for a strong electrical shock (1.05 mA), and, if BCAA treatment was stopped, cognitive function would again decline.^17^ Similarly, the water maze testing in the current study also detected that the pre-treated and pre-/post-treated groups performed better than post-treated animals. However, in addition, all treated groups (pre, pre/post, and post) performed better than the untreated group in both the 4- and 24-h memory probes, and these findings are consistent with the existing literature.^7,9,15–20^ For example, Cole and colleagues demonstrated that BCAAs restore cognitive function in an injured hippocampus without effecting the concentration of BCAAs in the contralateral normal hippocampus.

Moreover, BCAA-treated shams did not have elevated central nervous system levels of BCAAs, suggesting that the brain will pull BCAAs from the systemic circulation when in demand.^7^ Other examples are observed in two separate animal studies that examined the effects of BCAAs on sleep cycles post-TBI.^15,16^ Elliot and colleagues found that BCAAs normalized sleep cycles by restoring glutamate levels in the hypothalamus,^18^ and Lim and colleagues showed that BCAAs normalize electroencephalograms and sleep cycles after TBI.^19^ Last, two, severe TBI studies in humans demonstrated the protective effects of BCAA on cognitive function and the ability of BCAAs to pull TBI patients out of a vegetative state.^9,20^

A third area of support is that the GFAP data matched the cognitive assessment data. As with the cognitive results, the GFAP expression in the Pre + Post group was equivalent to what was observed in the shams. The general lack of a TBI-only effect on GFAP and IBA-1 may be attributable to the time the tissue was harvested at post-injury day 14. Cognitive deficits after TBI in rodents are known to self-resolve beginning at 2–3 weeks post-TBI, which is also consistent with human studies demonstrating resolution of TBI-induced deficits in visual memory, verbal memory, and reaction time at 2–3 weeks after TBI.^21–25^ Thus, tissue collection at an earlier time point after TBI may have shown an effect of TBI alone on GFAP and IBA-1. Conversely, it is highly plausible that the known effects of BCAAs on the GABA-glutamate shunt in astrocytes likely contributed to the astrocyte recruitment that we observed with pre-injury and post-injury BCAA treatment so that the balance of inhibitory and excitatory signaling would be better preserved.^19–22^ Future studies that incorporate microglial morphologies, biomarker or gene expression, and BCAA levels in the hippocampus and cortex would also provide better insights to these questions.

GFAP and IBA-1 provide a good indication of the number of astrocytes and microglia in a given area, but they do not provide information on the status of these cells. Cytokine levels and radioligand binding of the 18-kD translocator protein (TSPO) with PK-11195 do provide insight to activational status. Use of these markers was, unfortunately, not compatible with the design of this study. However, previous studies suggest that long-lasting (at least 3 weeks), TBI-induced increases in proinflammatory cytokines and TSPO binding in the brain can be reduced significantly by nutritional interventions.^21^ It is therefore possible that BCAA treatment had similar effects on these activational markers, and this assertion is supported by the normalization of astrocyte recruitment in animals treated with the Pre + Post BCAA combination.

Hospitalized TBI patients are typically managed by multiple service lines, including, but not limited to, neurosurgery, critical care, and trauma. Thus, it is important to emphasize the biochemistry of BCAA “nutrition” in the acute phase of TBI to clinicians. Leucine is unique, in that it is responsible for 30–50% of the nitrogen required for the synthesis of glutamate and glutamine in the brain.^25,26^ Leucine crosses the blood–brain barrier faster than any other amino acid, first passing into astrocytes, where it is swiftly transaminated, giving rise to glutamate and glutamine, as well as a branched-chain μ-ketoacid (KIC). The ketoacid is not oxidized at the same rate as it is produced. Instead, it is released from astrocytes to the neurons, which reverse the transamination process to reform leucine. This process also consumes glutamate and, conceivably, may function as a glutamate buffering system if levels of this excitatory amino acid become excessive. Leucine formed in neurons can be released back to astrocytes, thereby constituting a leucine-glutamate cycle that, like the glutamate-glutamine cycle, serves to shuttle nitrogen between astrocytes and neurons.^19,20,25,26^

A key biochemical fact is that leucine should not be given alone. It should instead be delivered in a 2:1:1 ratio of leucine/isoleucine/valine to avoid disrupting the temporal pattern of amino acid homeostasis.^27–29^ Further, leucine absorption is better through the oral route of administration versus intravenous, given that intravenous delivery rapidly saturates blood–brain barrier transporters.^18,20^ The dose required to provide neuroprotective effects in the current study was a human equivalent dose of 21 g/d of BCAAs with the 2:1:1: ratio and well below any of the tolerance studies.^30,31^ The 21-g dose was used to best reproduce the Aquilani and colleagues studies performed on humans at 19 g/d.

This study and Aquilani and colleagues both involved severe and moderate-to-severe TBI, thus requiring higher levels of BCAAs to combat increased levels of glutamate.^9,20^ Elango and colleagues analyzed the tolerable upper limit of leucine in young males and concluded that up to 1250 mg/kg was safe, which is >20-fold greater than the current U.S. Food and Drug Administration–recommended dose of 42 mg/kg.^31^ Matsumoto and colleagues determined that individual dosing of leucine, isoleucine, or valine was deleterious to overall amino acid homeostasis. However, when subjects were given a 6.3-g BCAA mixture (2:1:1 ratio), the other amino acids remained stable, and BCAA levels peaked after 30 min and remained elevated for ∼4 h. Thus, a BCAA mixture is safe for use in sports as a supplement or in medicine.^29^

## Conclusion

This is the first study demonstrating the neuroprotective effects of prophylactic BCAA in TBI. Previous findings in both animals and humans strongly support the neurorestorative benefits of BCAAs after TBI. It is unfortunate that BCAAs, though considered nutritional supplements, are not generally recognized for their beneficial effects. The results of this study demonstrated, through *in vivo* testing and post-mortem brain histological analysis, that BCAAs can protect the brain during a TBI as well as provide neurorestorative benefits after TBI. These results and the low risk of providing BCAAs to TBI patients should warrant further human clinical studies.

We believe the benefits of BCAA as a neuroprotective supplement, as well as neurorestorative, should not be overlooked and, with future research, could be a safe and effective approach to reduce concussion or TBI in military personnel and athletes in contact sports and treat patients who have suffered a TBI.

## Abbreviations Used

ANOVA: analysis of variance
BCAAs: branched-chain amino acids
GFAP: glial fibrillary acidic protein
IBA-1: ionized calcium-binding adapter molecule 1
RMANOVA: repeated-measures analysis of variance
TBI: traumatic brain injury
TSPO: 18-kD translocator protein
